# Challenges in the Management of Massive Carotid Body Tumor

**DOI:** 10.1155/2024/9963521

**Published:** 2024-04-29

**Authors:** Oluwapelumi Olusoga-Peters, Moses Ayodele Akinola, John Ifeanyi Nwadiokwu, Florence Oguntade

**Affiliations:** ^1^Olabisi Onabanjo University Teaching Hospital, Sagamu, Ogun, Nigeria; ^2^Department of Surgery, Ben Carson College of Health and Medical Sciences, Babcock University, Ilishan Remo, Ogun, Nigeria; ^3^Department of Anatomic Pathology, Ben Carson College of Health and Medical Sciences, Babcock University, Ilishan Remo, Ogun, Nigeria

## Abstract

The carotid body paraganglioma is a rare benign neoplasm arising from the chemoreceptor cells of the carotid bulb. The carotid body has the largest collection of paraganglia in the head and neck with 60–70% of head and neck paraganglioma. Paraganglia are clusters of cells originating from the neural crest with histological and cytochemical characteristics of neuroendocrine cells. It is mostly asymptomatic in early presentation but become symptomatic and difficult to manage when the tumor is large. We present a case of a 26-year-old male who presented with a painless, pulsatile, progressively increasing left lateral neck swelling of 5 years duration with Shamblin IIIa. The diagnosis of the tumor was confirmed based on clinical features, histology, and radiological findings. We had difficult surgical dissection of the tumor with neurovascular damage.

## 1. Introduction

The carotid body is found on the medial aspect of the carotid bifurcation as the largest collection of paraganglia in the head and neck. Carotid body tumors (CBTs) are rare neuroendocrine neoplasms which are near the carotid bifurcation within glomus cells derived from the embryonic neural crest [1]. They are also called paragangliomas or chemodectomas. CBTs are chemoreceptor tumors accounting for 65% of all head and neck tumors [2].

Incidence of CBTs is less than 1-2 in 100,000 [2], more common in females than males (1.9 : 1). The peak age group is between 50 and 70 years of age. Bilateral tumors account for approximately 10% of the cases. Also, malignant tumors account for below 10% of CBTs [3, 4].

The Shamblin research group in 1971 introduced a classification system based on the relationship of the tumor with the carotid arteries to determine the resectability of the tumor. In type I, the tumors are small lesions and do not splay the carotid bifurcation. Type II tumors are larger and significantly splay the carotid bifurcation but do not circumferentially encase the carotid arteries. Type III tumors are large, encapsulate the internal or external carotid arteries, and often incorporate the adjacent cranial nerves. Modified shambling classification later divides type III into **a** and **b** where **a** is when the tumor only encases the carotid vessels and **b** is when the mass is infiltrating the vessel wall. Type IIIb is associated with more perioperative neurovascular complications and complex surgical procedures [5–7].

Majority of carotid body tumors are asymptomatic and usually noticed first as incidental findings on physical examination of the neck or during radiological studies. However, large tumors can produce symptoms secondary to pressure effect and local invasion of the surrounding tissue such as pain, difficulty with breathing, dysphagia, and autonomic dysfunction [8, 9]. The most involved nerves are hypoglossal, glossopharyngeal, vagus and the sympathetic chain [10].

A case of bilateral carotid body tumor is presented. The patient is a 26-year-old male who consulted our otorhinolaryngology clinic with a left-sided neck swelling of 5 years. The clinical picture, radiological investigations, and surgical treatment are described below.

## 2. Case Report

The patient is a 26-year-old male who presented to our clinic with a five-year history of a left-sided neck mass. The mass initially started as a small lump and continuously increased in size reaching current size at presentation two years ago.

The mass was non-discharging and not painful.Patient developed noisy breathing, dysphagia, difficulty with breathing and recurrent cough two months before presentation.However, there was no history suggestive of release of catecholamine.

Physical examination of the mass revealed a scar on the surface and a small ulcer that was granulating due to previous attempt at biopsy in the referral centre; it is a 10 cm by 8 cm mass that was firm in consistency, non-tender, non-pulsatile, and non-mobile with well-defined edges; there was no differential warmth, and it was attached to the overlying skin. There was a small pulsatile mass felt on the right side of the neck. There was no palpable cervical lymph node enlargement; oropharyngeal examination showed a left-sided mass protruding into the oropharynx behind the tonsil, approaching the midline covered with normal mucosa. Laryngoscopy done revealed mass deviation of the larynx to the right with reduced movement of the ipsilateral vocal cord.

**Figure 1:**
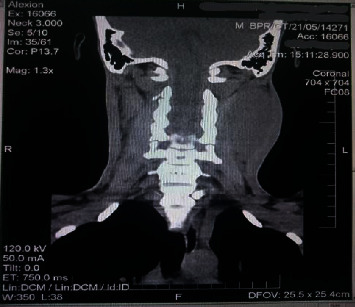
Coronal view of CT neck showing an isodense mass with well-defined edges on the left.

CT scan of the neck showed large avidly enhancing highly vascular masses in both carotid spaces (right 26 mm by 29 mm, left 86 mm by 75 mm). The masses were splaying the internal and external carotid arteries, indenting the internal jugular vein with associated narrowing of the nasopharyngeal and oropharyngeal air column. No enlarged cervical lymph node was seen (Figure 1).

**Figure 2:**
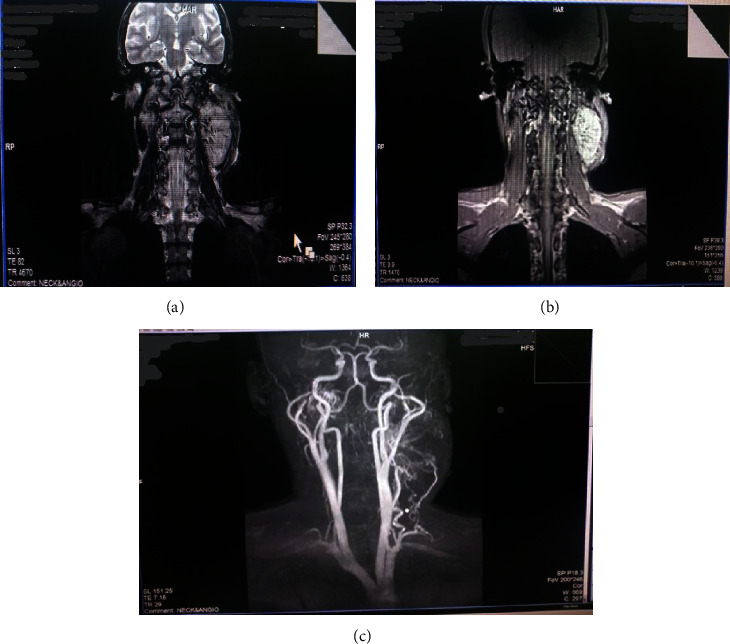
(a, b) An MRI image of the same mass. (c) MRI angiography demonstrates a highly vascular mass splaying the internal and external carotid arteries.

MRI showed a well lobulated lesion in the left carotid space. The mass had a slightly higher signal on T2W and lower signal on non-contrast T1W with strong enhancement on contrast-enhanced T1W. MRI angiography also revealed splaying of the carotid vessels at the carotid bifurcation with multiple feeders (Figures 2(a)–2(c)).

**Figure 3:**
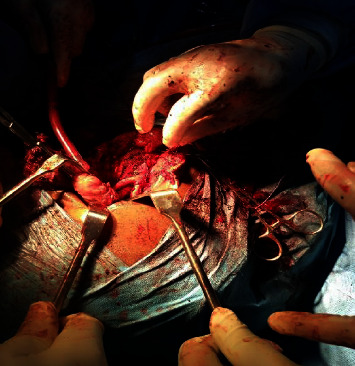
Intraoperative picture of the bifurcation of the common carotid artery following tumor excision.

Diagnosis of CBT was made, and the patient was worked up for excision of the left tumor. The right side was planned for later date to avoid bilateral injury to carotid arteries and nerves. Informed and well understood consent was obtained including the possibility of sacrificing involved cranial nerve and reconstruction of carotid vessel if infiltrated. Surgery was done with the assistant of a cardiothoracic surgeon. General anaesthesia via an orotracheal intubation was done. Following routine skin preparation and draping, the left side of the neck was explored by a Schobinger incision. A hemorrhagic mass, firm in consistency with significant bleeding on contact due to multiple varicose vessels, was seen all over it (Figure 3). There was significant blood loss during dissection with estimated blood loss of 6 liters. It was firmly attached to the underlying big vessels and nerve (left internal jugular vein, left external and internal carotid arteries, and vagus nerve) and overlying muscle making the dissection very difficult. The left internal jugular vein, vagus nerve, and anterosuperior part of the sternocleidomastoid muscle were excised alongside the mass due to the extent of the adherence to these structures. The patient was transfused with 8 pints of blood and 4 units of fresh frozen plasma.

**Figure 4:**
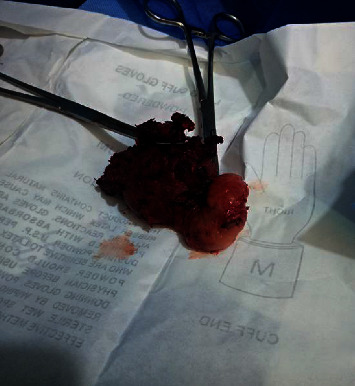
The picture of the specimen resected.

The specimen measured 12 cm by 14 cm and extended from the para-pharyngeal space to the upper part of the common carotid artery (Figure 4).

Following total excision of the tumor, the wound was approximated and the skin was closed with interrupted stiches.

**Figure 5:**
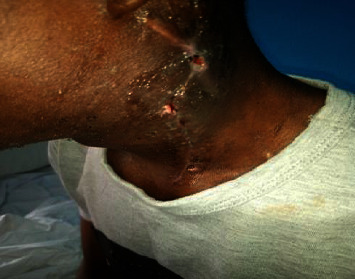
Two weeks post-op picture showing the post-op scar.

The patient was transfused with eight units of whole fresh blood and four units of plasma during the surgery and the immediate postoperative period. Estimated blood loss was 7,350 mls. Post-op/posttransfusion PCV was 37.5%. Neck drain was removed after two days. Brownish effluent which soaked the neck dressing developed nine days post-op when part of the sutures was removed and lasted for two days, and the patient was discharged on the thirteenth day post-op. Though the patient had resolution of respiratory distress and stridor immediately after surgery, he however complained of dysphagia and aspiration afterward due to the vagus nerve damage which has improvement significantly. He is alive with a healed scar along the line of the skin incision (Figure 5). He was advised to present for surgery excision of the right-side CBT but defaulted.

**Figure 6:**
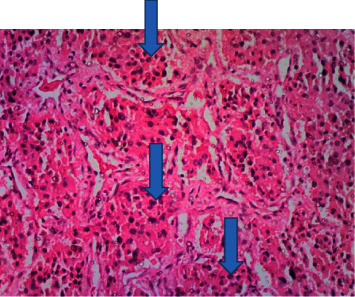
Histological section showing nested packets of mild to moderately pleomorphic tumor cells (arrows) having hyperchromatic central nuclei and moderate eosinophilic, faintly granular cytoplasm consistent with the features of paraganglioma (H&E ×40).

**Figure 7:**
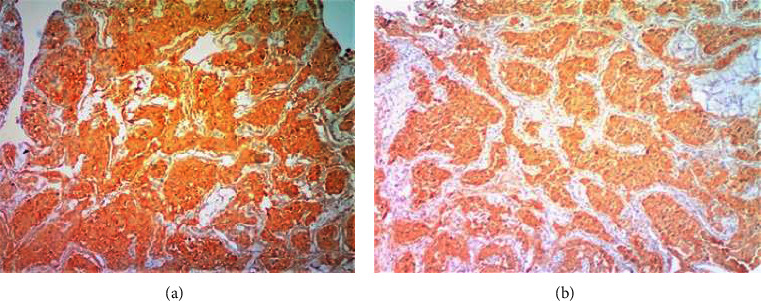
(a) Chromogranin, (×40) immunohistochemical stain (3^+^) in about 80% of the tumor cells in keeping with a diagnosis of paraganglioma. (b) Synaptophysin, (×40) immunohistochemical stain showing positive diffuse cytoplasmic staining (3^+^) in about 95% of the tumor cells in keeping with a diagnosis of paraganglioma.

Histologically, fragments of tissue with tumor cells disposed predominantly in nested packets and separated by extensive delicate vascular network giving an organoid arrangement (zellballen pattern) were seen. The component cells had mild to moderately pleomorphic and hyperchromatic central nuclei, coarse chromatin pattern, and moderate eosinophilic, faintly granular cytoplasm. Also seen were areas of stromal hyalinization, focal necrosis, and hemorrhage (Figure 6). The immunohistochemisty result revealed reactivity of tumor cells to chromogranin, neuron-specific enolase, and synaptophysin stains (Figure 7).

## 3. Discussion

The carotid body is the largest collection of paraganglia in the head and neck accounting for 65% of tumors in the head and neck [2, 3]. The tumor is found in the carotid space. The carotid space runs from the lower border of the jugular foramen all the way to the aortic arch, defined by the layers of the deep carotid fascia. The space includes the supra- and infrahyoid regions [11].

The earliest description of carotid body was by von Haller in 1743. He described it as a “reddish-brown, well circumscribed, specialized organ, located in the adventitia of the carotid bifurcation, supplied by the feeding vessels from the ascending pharyngeal branch of the external carotid artery, and innervated by the glossopharyngeal and vagus nerves” [12].

It functions as a chemoreceptor organ, and it is stimulated when there is acidosis, hypoxia, and hypercapnia resulting in autonomous control of heart rate, blood pressure, temperature, and respiration by increasing sympathetic flow [13].

CBTs are mainly seen in the 50 and 70 years of age, more in women than men (1.9 : 1); however, in our case, the patient is a 26-year-old male with bilateral neck tumor [2, 3].

The location of CBTs has made it one of the challenging neck tumors to manage in head and neck surgery. They are mostly benign though about 10% have been reported as malignant [1, 2], thus the need to ensure complete resection during surgery as done in our case.

Bilateral cases of CBTs have been reported to be mostly familial (31.8%) and multifocal in nature while the sporadic cases are rarely bilateral and are less multifocal in nature. Our patient also presented with bilateral CBT with positive family history as documented in several familial cases [14].

The carotid body is between 2 and 6 mm in diameter. However, in people living in high altitude, it is often larger. Saldana et al. reported increased incidence of the tumor in Peruvian patients who live in high altitudes and concluded that hypoxia was the primary stimuli leading to carotid body hyperplasia. Though several studies have associated living in high altitude and COPD with neoplasm of the carotid body, there is no history of such in our patient [15].

Dysphagia, hoarseness, and symptoms due to compression have been reported in large tumors due to the close relationship of the tumor and the CN X-XII and its extension to the pharynx as seen in our patient [8, 9]. The most involved cranial nerve is vagus nerve, located in between the IJV and the carotid artery.

Some patients with CBT give history suggestive of neuroendocrine secretion causing catecholamine-related symptoms such as fluctuating hypertension, flushing, palpitation, and tachycardia. However, our patient did not present with these clinical features [16].

CBTs are slow growing, non-tender, and lateral neck mass located in the carotid bifurcation at the level of hyoid bone and anterior to the sternocleidomastoid muscle. It is mobile in lateral plane but has limited mobility in vertical plane (Fontaine's sign); occasionally, the tumor may transmit the carotid pulse or demonstrate a bruit/thrill. Our case is also a non-tender lateral mass, in the usual location, and it is not mobile on all planes possibly due to previous attempt at biopsy leading to adhesion to overlying structures but has a bruit on auscultation [17].

Size of the tumor has a great importance in the clinical manifestation and treatment. While small tumor classified as Shamblin I can be easily dissected from the vessels without no injury, groups II and III which are closely associated to the carotid vessels are more difficult to dissect without injury to the vessel as seen in our case [5–7].

Several radiological investigations to support the diagnosis of CBTs include Doppler ultrasonography, CT scan, and MR angiography. CT is helpful in assessing the tumor location, size, and invasiveness of the tumor while MR angiography will assess the blood vessels that are feeding the tumor. MR angiography and CT scan were done in our patient which were helpful in diagnosis and preoperative preparation of our patient, revealing the location, extent of the tumor, and its relationship to contiguous structures, feeding vessels, and the hypervascularity of the mass [18, 19].

The Doppler ultrasonography is a non-invasive imaging modality that is widely used to screen for CBT in familial cases. Other investigations to rule out functional type of CBT were not done in our patient due to lack of history suggestive of secretion of catecholamine [18].

Biopsy as a diagnostic method is contraindicated because of the hypervascularization and proximity of the tumor to important structures like carotid vessels and the last four cranial nerves. This can be complicated with massive hemorrhage, dissemination of tumor cells, pseudoaneurysm formation, and carotid thrombosis [6, 7].

Preserving injury to the carotid vessels and the surrounding nerves is the primary concern during surgical resection of CBTs. Injury to these structures and high mortality have been documented in Shamblin's III tumors and tumors that are more than 5 cm in size. Therefore, it is better to have an early diagnosis and early resection is advocated to avoid associated nerve(s) injury and vascular damage during surgery. The size of the mass in our case was more than 5 cm in size and it is a grade III shambling classification as it was encasing the vessels and nerve leading to resection of the vagus nerve and significant hemorrhage [5, 20, 21].

Treatment of carotid body tumor is mainly surgical even in Shamblin class III [22]. However, radiotherapy has been advocated in residual tumor, unresectable giant tumors, and in malignant cases, with lymph node metastasis. Though our patient had a giant tumor, we were able to completely excise the tumor and the histopathological report was benign, negating the need for radiotherapy [23].

The choice of incision largely depends on the extent of the tumor. They include curvilinear incision along the midpoint of the tumor, hockey stick incision along the anterior border of sternocleidomastoid muscle, and a modified radical neck T incision (Schobinger incision) in very high and extensive tumors. The Schobinger incision was used in our patient due to the giant size of the tumor [23].

Bleeding is an important complication during surgical excision of the tumor and has been reported as massive especially in large tumor. To reduce significant bleeding, embolization of the feeding vessels before surgery has been used in large tumors which has been reported to reduce intraoperative blood loss but has been found not to reduce rate of nerve injury [24]. Embolization has also been reported to be associated with inflammation leading to damage of arterial wall, thereby making periadventitial dissection difficult which can be prevented by placement of a stent in external carotid artery [21, 25, 26]. However, where it is available embolization has been advocated for tumors classified as Shamblin's class III [27]. Embolization was not done in our case due to lack of expertise in our centre. The dissection of our patient was done with the help of diathermy and ligature machine which was helpful in reducing the blood loss from feeding vessels.

The use of subadventitial dissection of the tumor from the carotid arteries can also minimize blood loss during surgical excision of large tumor [20]. We were able to use subadventitial dissection to separate the tumor from the carotid vessels in our case with minor vascular damage that was immediately repaired. Balloon occlusion of the internal carotid artery could be done to assess if the brain can tolerate occlusion of the vessel such as clamping and shunting during surgery [28]. In worse scenario in some Shamblin III, tumors are removed along with resection of the bifurcation of the carotid and vascular reconstruction of the internal carotid artery is done [29]. In our case, the common carotid artery was intermittently clamped, and placing an ICA shunt was not feasible in our case because the mass was totally encasing the ICA from its origin in the neck to the base of the skull.

Reports of several studies revealed that the rate of complications following surgical removal of CBT is different. However, it is consistent that the modern surgical technique has reduced the risk of stroke following resection of CBT, but the incidence of cranial nerve injury is still high. Majority of the nerve injury will recover within six months but some of the cases are said to have permanent neurological deficits. Our patient has vagus nerve damage which has not fully recovered six months postoperatively [5, 30, 31].

Our histopathological findings are also in support of the classical histology of CBT which is highly vascularized with clusters of cells in between the many capsules. A second smaller tumor was seen in the right carotid space on radiograph, and the patient was advised to present at a later period for the excision of the second tumor to avoid uncontrollable blood pressure postoperatively [32].

## 4. Conclusion

Operating giant CBT is challenging due to hypervascularity of the tumor from several kinds of neovascularity of major vessels leading to massive hemorrhage. The proximity and adhesion of the tumor to major vessels and nerves commonly result in damage of the nerves and vessels. Early diagnosis and treatment of CBT should be encouraged to make surgical excision easier with minimal or no complications.
